# Uncovering Metal-Decorated TiO_2_ Photocatalysts for Ciprofloxacin Degradation—A Combined Experimental and DFT Study

**DOI:** 10.3390/ijms252111844

**Published:** 2024-11-04

**Authors:** Marija Kovačević, Marija Simić, Sanja Živković, Miloš Milović, Ljiljana Tolić Stojadinović, Dubravka Relić, Dragana Vasić Anićijević

**Affiliations:** 1 Vinča Institute of Nuclear Sciences-National Institute of the Republic of Serbia, University of Belgrade, Mike Petrovića Alasa 12-14, 11351 Belgrade, Serbia; 2 Institute of Technical Sciences of SASA, Knez Mihajlova 35/IV, 11000 Belgrade, Serbia; 3 Innovation Centre of the Faculty of Technology and Metallurgy, Karnegijeva 4, 11000 Belgrade, Serbia; 4 Faculty of Chemistry, University of Belgrade, Studentski Trg 12-14, 11158 Belgrade, Serbia; dradman@chem.bg.ac.rs

**Keywords:** photocatalysis, ciprofloxacin, rutile TiO_2_, DFT calculations, metal decoration

## Abstract

Optimization of the efficiency of the photocatalytic degradation of organic and pharmaceutical pollutants represents a matter of fundamental and practical interest. The present experimental and DFT study deals with evaluation of OH radical binding energy as a simple computational descriptor of the catalytic activity of *d-*metal-decorated TiO_2_ photocatalysts for the photodegradation of the widely used antibiotic ciprofloxacin. Five *d-*metals commonly used in catalytic materials (Zr, Pt, Pd, Fe, and Cu) were deposited on the TiO_2_ surface, and the obtained photocatalysts were characterized experimentally (XRPD, ICP-OES, and SEM) and theoretically (DFT). Attention was also paid to the mechanistic insights and degradation byproducts (based on UV-Vis spectrometry and LC/MS analysis) in order to obtain systematic insight into their structure/performance relationships and confirm the proposed model of the degradation process based on OH radical reactivity.

## 1. Introduction

The usage of antibiotics for human and animal treatment is increasing, persistent, and ubiquitous [1,2]. Ciprofloxacin is a widely used compound from the fluoroquinolone class. Interest in the investigation of its removal from the environment has both practical (high abundance), and fundamental (easily tracked model molecule, considerable chemical inertness) significance [3,4,5,6,7,8].

UV-based photocatalysis, among other advanced oxidation process methodologies, is a well-established method for the removal of antibiotics and organic pollutants in general [9,10,11,12,13]. The main advantages of photocatalytic degradation are low cost, low power consumption, and simple operability because it is performed at room temperature and pressure and uses atmospheric oxygen for oxidation. The process is considered environmentally friendly because it does not require additional chemicals and the catalysts are generally considered reusable [14,15,16]. Major disadvantages include the small size of the reactors (laboratory use), limited potential to utilize sunlight, short lifetime of photo-generated electrons, and potential toxicity of byproducts [17]. Research conducted so far has shown that semiconductors like metal oxides (TiO_2_, SnO_2_, Fe_2_O_3_, WO_3_, ZnO, Ag_3_O_4_, Nb_2_O_5_, CeO_2_, MoO_3_, ZrO_2_), metal sulfides (ZnS, CdS), and g-C_3_N_4_-based materials are promising photocatalysts [16,18,19,20]. The wide range of uses for TiO_2_ can be attributed to its excellent optical and electronic properties, high chemical and biological stability, low cost and availability, non-toxicity, resistivity towards corrosion, and eco-friendliness [21,22,23,24], despite its large band gap and minimal utilization of the UV area of sunlight [25]. Some experimental strategies used to reduce band gap are element doping, coupling with other semiconductors, synthesis of nanostructures with particular morphologies, surface sensitization using organic dyes, and the use of metal-based structures [26,27,28]. Besides the metal doping modification of TiO_2_, research also shows that the deposition of a thin layer of noble metals (Au, Ag, Pt, and Pd) onto the surface of TiO_2_ can improve photocatalytic properties and efficiency. Although there is a significantly smaller number of studies on the metal decoration of TiO_2_ compared to ion doping, the researchers agree that metal atoms impede electron–hole recombination by trapping electrons. This approach is further closely bound to single-atom catalysis and its remarkable perspectives [29,30,31,32,33,34].

The involvement of such a large variety of materials and sophisticated structural modifications in photocatalysis implies a need for the systematic understanding, control, and prediction of material properties. Identification of simple computational descriptors that determine catalytic performance accelerates the identification of materials with potentially high photocatalytic efficiency. DFT descriptors are well established in the screening of electrocatalytic materials [35,36]. However, to our knowledge, this approach has not been used to describe photocatalysts, despite similarity between the molecular backgrounds of the processes [37]. From the perspective of a fundamental understanding of the photocatalytic process, the correlation between bandgap and performance is well explored. On the other hand, a small number of studies still deal with surface phenomena in photocatalysis, including the chemical behavior of the reaction species involved in the process.

The aim of this study was to establish potential theoretical descriptors of the activity of a *d*-metal-decorated TiO_2_ photocatalyst by calculating the OH radical binding strength on the surface and analyzing its effects on the catalytic process. A set of five *d-*metals commonly used in the preparation of photocatalysts, each with different chemical and electronic properties (Zr, Pt, Pd, Fe, and Cu), was deposited on TiO_2_ surface, and the obtained materials were employed for the photocatalytic degradation of ciprofloxacin. The photocatalysts were modelled using density functional theory (DFT) calculations and characterized experimentally (XRPD, SEM, ICP-OES) in order to verify their morphostructural features and chemical composition. Ciprofloxacin degradation was monitored by UV-Vis spectrometry. Additional attention was paid to the degradation products and the fate of the ciprofloxacin when using the most efficient of the prepared photocatalysts. LCMS tracking was used to verify the proposed structure/performance relationships and provide additional mechanistic insights.

## 2. Results and Discussion

### 2.1. Establishing OH Radical Binding Energy as a Theoretical Descriptor of Photocatalytic Reactivity

Establishment of valid theoretical descriptors comprises successful correlation of the proposed calculable catalyst properties with experimentally measured performance (i.e., degradation process rate and efficiency). In this work, the identification of potential theoretical descriptors is based on the well-established mechanism of the photocatalytic reactivity. The first step in the photocatalytic process is the formation of an electron–hole pair:

(1) $$hv\to h^{+}+e^{-}$$

The energy required for this step is the band gap energy. Regarding potential theoretical descriptors, this step can be evaluated if the DFT-calculated band gap energy E_gap_ obtained from projected density of states (PDOS) calculations is correlated with experimentally measured catalytic performance.

The subsequent reaction steps involve the formation, interaction, and desorption of OH radicals. According to the approach proposed by Tran et al. [38], after the surface site (*) is affected by photoexcitation, the immediate water splitting reaction is assumed to occur, so the amount of OH radicals generated on the surface is equivalent to the total number of photoexcited sites:

(2a) $$\left[*]=[{OH}^{*}\right]$$

The next step involves the reversible adsorption of the reactant R:

(2b) $$R+{OH}^{*}⇌R-{OH}^{*}$$

Subsequently, adsorbed R-OH* form proceeds to desorption and degradation, leaving a free active site to continue the process:

(2c) $$R-{OH}^{*}\to Q+*$$

The contribution of the steps (2b) and (2c) can be evaluated if the DFT-calculated adsorption energy of OH radical (E_ads,OH_) is correlated with experimentally measured catalytic performance. Based on the above considerations, we selected DFT-calculated E_ads,OH_ and band structure (PDOS) to be evaluated as potential reactivity descriptors of TiO_2_(M) photocatalysts.

For DFT calculations, TiO_2_(M) catalysts were represented as the TiO_2_(110) surface slabs with adsorbed metal atoms at a coverage of 0.5 ML, since TiO_2_(110) is the most abundant plane in all samples, according to the XRD measurements (for details, see Section 2.4.1). Optimized model structures with adsorbed metals are represented in Table 1.

**Figure 1:**
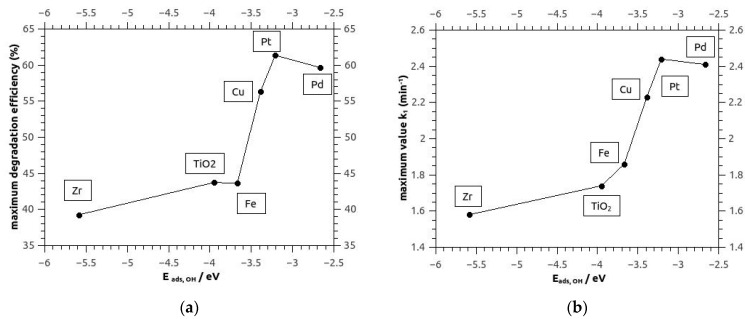
Relation between DFT-calculated E_ads,OH_ and (**a**) maximum degradation efficiency and (**b**) maximum degradation rate. In DFT input geometries, OH radical is put on the metal top site. The maximum degradation efficiency (rate constant) was selected as having the largest degradation extent (or highest rate constant) among the 0.5%, 1%, and 2% TiO_2_(M) catalysts.

The adsorption energy of the OH radical on the M-top site is calculated on the geometrically optimized TiO_2_(M) surfaces. On the optimized TiO_2_ surface, Fe and Cu occupy O-top sites while Zr, Pd, and Pt are in the hollow position. E_ads,OH_ is correlated with selected experimental degradation performance indicators, which are the maximal degradation efficiency and maximal rate constant for each TiO_2_(M) system. The maximum degradation efficiency (rate constant) was selected as having the largest degradation extent (or highest rate constant) among the 0.5%, 1%, and 2% TiO_2_(M) catalysts. Details of the degradation experiment are provided in Section 2.3. The results are shown in Figure 1.

From Figure 1, it is obvious that both degradation rate and efficiency increase with a decrease in the adsorption strength of the OH radical on the investigated surface (weaker OH radical binding means more-efficient degradation). The increase in degradation efficiency and reaction rate upon a decrease in DFT-calculated E_ads,OH_ on the metal-decorated TiO_2_ surface confirms the crucial role of chemical surface properties and OH radical binding strength in reactivity for ciprofloxacin degradation. According to the observed trend, the weaker binding of OH radical accelerates the desorption (step (2c)). Moreover, according to previous literature data on similar processes involving the adsorption and desorption of oxygen-containing species [35,36], one could propose that the obtained dependence is a branch of a broader volcano-shaped correlation, implying that there probably would be an optimal value of OH binding energy for the best degradation performance. If the binding energy of OH radicals further decreased below the optimal value, one could expect a decrease of degradation performance due to the thermodynamical inability of the metal active site to split water.

**Figure 2:**
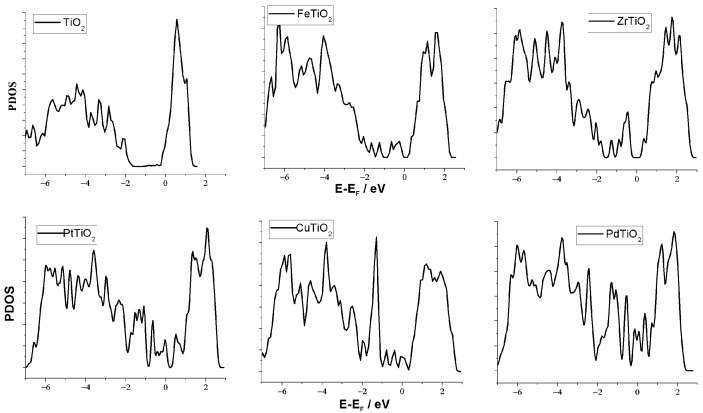
PDOS structures of bare TiO_2_(110) and TiO_2_(110) with deposited metals Pt, Pd, Zr, Cu, and Fe.

To evaluate PDOS structure as a potential catalytic descriptor, DFT-calculated PDOS structures were represented for bare TiO_2_ and all TiO_2_(M) surface models (Figure 2).

In the majority of studies dealing with photocatalytic degradation by TiO_2_(M) materials, the proposed improvements compared to bare TiO_2_ are ascribed to the modification of the optical properties of the catalyst (reduction of band gap energy leading to the more-efficient generation of active sites) [39]. However, using the here-proposed model surfaces with a rather high TiO_2_(M) ratio and a metal coverage of 0.5 monolayer (ML), no reliable data on the systematic effect of metal deposited to the gap structure can be claimed. In our model, the impact of metal introduction on PDOS is similar for all adsorbed metals, involving new PDOS states appearing in the gap area upon metal deposition. Our model generally suggests that the catalyst surface locally (in the vicinity of the large metal atoms such as Pd and Pt or at high coverages of deposited metals) obtains a metallic character.

### 2.2. Additional Insights into TiO_2_(M) Structure: (Pt)TiO_2_ and (Pd)TiO_2_ as Single-Atom Catalysts

For the experimental measurement of degradation rate and efficiency, each TiO_2_(M) catalyst was prepared at three input metal concentrations (0.5, 1, and 2 at% of metal). The amount of metal deposited on the TiO_2_ surface was measured experimentally by ICP-OES and EDS. ICP-OES data were further used to estimate metal surface coverage (see Supplementary Material S1 for detailed calculations). The results are represented in Table 2.

Despite very low coverages of Pd and Pt—only 1–2 atoms nm^−2^, even at the highest input metal concentrations of 2%—these photocatalysts also exhibit the highest photocatalytic performance among all the catalysts investigated. The results are corroborated by EDS spectra (Figure S1) and EDS maps (Figure S2). These emphasize the contribution of single-atom sites as the crucial points where OH radicals are efficiently generated and desorbed during the photocatalytic process. On the other hand, such results impose a need to discuss the factors that determine the affinity of the metal to bind on the surface. Toward this goal, the literature values of metal cohesive energies (E_coh,lit_) are correlated with DFT-calculated values (E_M-TiO2_) and experimental data on metal coverage. The higher E_TiO2-M_ (absolute value) is compared to E_coh_, the more stable deposit is expected to be. When E_coh_ is higher than the absolute value of E_TiO2-M_, the formation of metal agglomerates is expected, and vice versa: when the absolute value of E_TiO2-M_ is higher than E_coh_, the formation of stable deposits and thin layers is probable.

**Figure 3:**
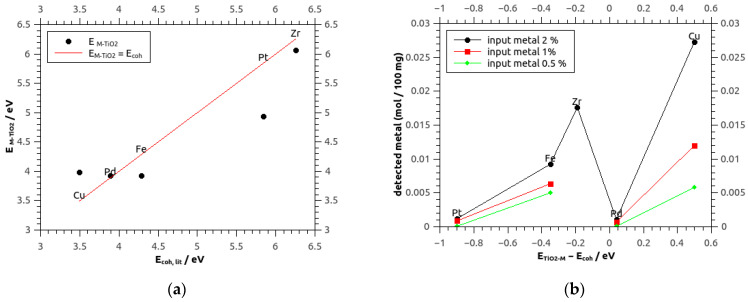
(**a**) The dependence of DFT-calculated E_M-TiO2_ vs. literature E_coh,lit_ on TiO_2_(110) for investigated metals at a surface coverage 0.5 ML. Absolute values are shown for clarity. The red line represents E_M-TiO2_ = E_coh_ dependence. (**b**) The dependence of experimentally detected metal level (expressed as mol/100 mg) in the catalyst samples on the difference E_TiO2-M –Ecoh,_ for three input metal levels (0.5, 1, and 2%).

The correlation between the literature values of cohesive energies [40] and calculated E_M-TiO2_ are provided in Figure 3a. Figure 3b represents the correlation of E_TiO2-M_ − E_coh_ vs. ICP-OES detected molar concentration for the three input metal levels (0.5, 1, and 2 at%) for each metal. The graph shows the trend of the increased amount of deposited metal with the increase in E_TiO2-M_ − E_coh_ and the initial metal concentration (with the exception of Pd, a very low amount of which was deposited despite the positive value of E_TiO2-M_ − E_coh_).

The results generally confirm that the highly cohesive metals (such as Pt) will be deposited at very low coverages and will weakly interact with the support, while those with lower cohesivity and high affinity for binding to TiO_2_(110) (such as Cu) are expected to form thin layers. However, independent of their surface states, the investigated metals contribute to the catalytic activity of the materials in the same manner, via the above-proposed modification of OH radical reactivity. This effect is most prominent in the case of deposited Pt and Pd, single atoms with remarkable power for water splitting.

### 2.3. Measurements of Photodegradation Efficiency

**Figure 4:**
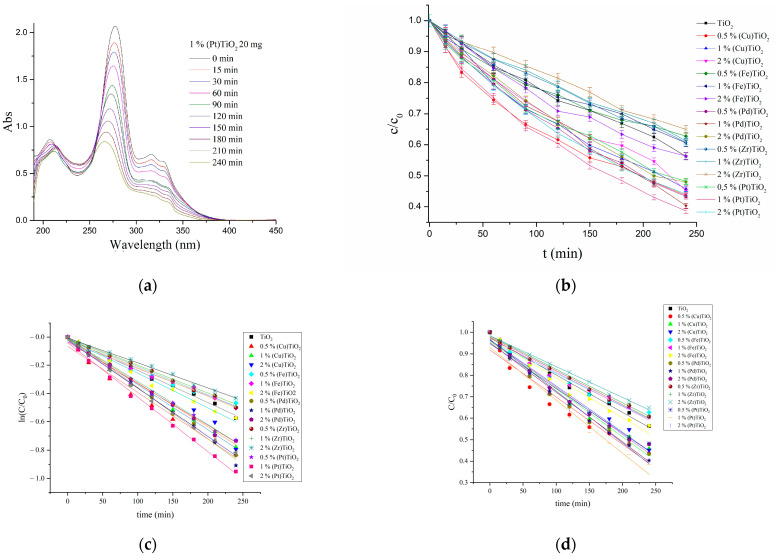
UV Vis adsorption spectra of ciprofloxacin degradation on 1%(Pt)TiO_2_ (**a**), degradation curves (**b**), and their linearized pseudo-first-order (**c**) and pseudo-zero-order (**d**) fits of ciprofloxacin commercial solution for the infusion within 240 min.

Prepared photocatalysts—Pt-, Pd-, Zr-, Cu-, and Fe-modified TiO_2_—were applied for the degradation of the ciprofloxacin over a duration of 240 min. The representative UV-Vis adsorption spectra of the degradation process with 20 mg 1%(Pt)TiO_2_, degradation curves, and their pseudo-first-order and pseudo-zero-order fits are provided in Figure 4. All degradation spectra are provided in the Supplementary Material, Figure S3a.

The UV-Vis spectra confirm that the characteristic absorption maximum of ciprofloxacin is at about 277 nm, which decreases with time due to the photodegradation. The appearance of well-defined isosbestic points at about 215 and 260 nm indicate that the equilibrium process involving the depletion and formation of compounds occurs. The gradual hypsochromic shift of the absorption maximum from 277 nm points to the formation of degradation products that are structurally similar to ciprofloxacin but with lower ability to donate an electron. The comparison with irradiated blank spectra (Supplementary Material, Figure S3b) confirm that the process rate is negligible in the presence of UV light without the catalyst, and the observed degradation originates principally from the processes on the catalyst surface.

In this work, the degradation extent was measured by fitting of concentration depletion (C/C_0_) by pseudo-first order

(3a) $$C=C_{0}\cdot e^{-k_{1}t}$$

and pseudo-zero order

(3b) $$C=C_{0}-k_{0}\cdot t$$

where C_0_ is the ciprofloxacin concentration at time t = 0, and k_1_ (min^−1^) and k_0_ (mol L^−1^ min^−1^) are a pseudo-first-order and pseudo-zero-order rate constants, respectively.

Calculated pseudo-first- and pseudo-zero-order rate constants and the final degradation efficiencies over 240 min are given in Table 3.

The observed increase in catalytic activity upon the deposition of *d*-metal is reflected in the increase in the efficiency of ciprofloxacin degradation from 43 to 61%, as well as in the increase in the rate constant of the degradation reaction by up to 1.70 times, depending on the type of *d*-metal deposited on the TiO_2_ surface.

The R^2^ values confirm a better correlation for pseudo-first-order than pseudo-zero-order kinetics for all materials except (Zr)TiO_2_, where R^2^ values are similar for both types of kinetics. The better applicability of the pseudo-first- compared to pseudo-zero-order kinetics, in light of considerations from [38], points to the adsorption/desorption process as the rate-determining step, which is also in a good agreement with the proposed model and the DFT predictions regarding the crucial role of OH radicals in desorption.

**Figure 5:**
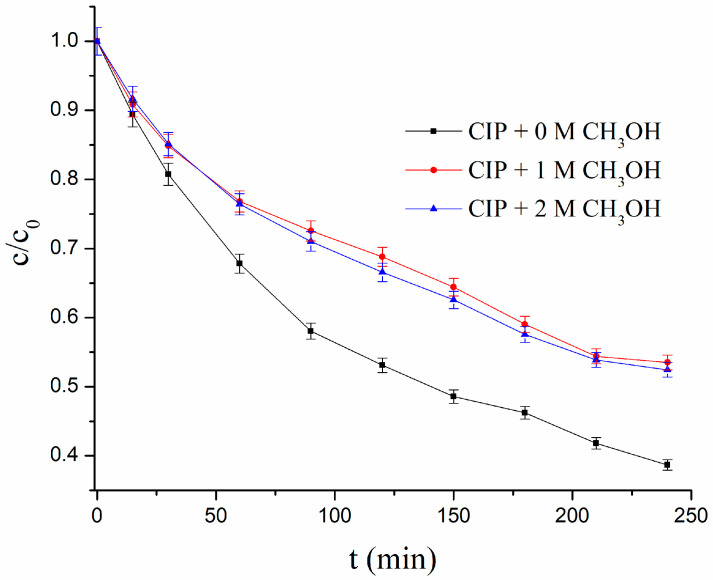
The degradation curve of ciprofloxacin on 1%(Pt)TiO_2_ in the presence and absence of methanol.

To confirm the contribution of OH radicals to the oxidation process, the degradation experiment on the most efficient 1%(Pt)TiO_2_ photocatalyst was performed in methanol. In the presence of methanol, OH radical catching contributes to the reduction in reaction rate and efficiency. The results are provided in Figure 5.

Figure 5 confirms that the degradation efficiency has been reduced from 61.5% to 46.5%, followed by a reduction in the degradation rate constant from 3.87 to 2.51 × 10^−3^ min^−1^.

### 2.4. Morpho-Structural Characterization of Prepared Photocatalysts

#### 2.4.1. XRPD Analysis of Catalyst Powders

**Figure 6:**
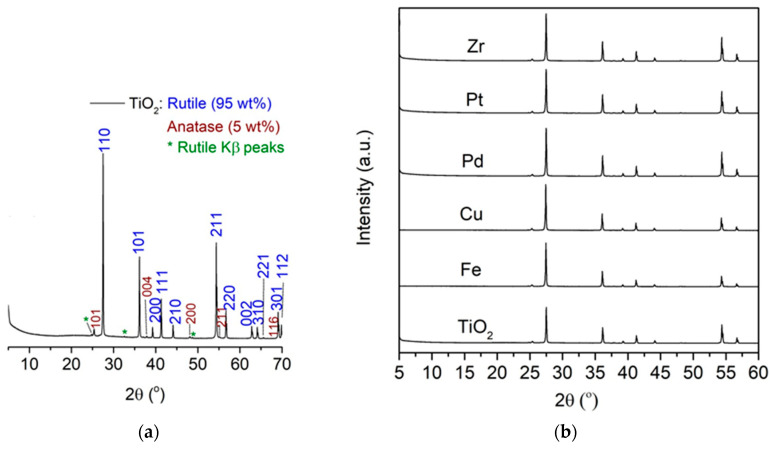
XRPD pattern of TiO_2_ support powder (**a**) and the metal-deposited powders (**b**).

The XRPD pattern of the prepared TiO_2_ powder is presented in Figure 6a. All reflections have been indexed within the rutile (PDF card no. 21-1276) and anatase (PDF card no. 21-1272) phase. Based on the relative ratio of intensities of all peaks and I/I_cor_ (a.k.a. RIR [41]) coefficient values for the proposed PDF cards, the fraction of anatase has been estimated to be no more than 5 weight%. The XRPD patterns of the respective metal-decorated TiO_2_ powders are displayed in Figure 6b. There are no additional reflections that have been detected, probably due to the very small loading of deposited metal and/or poor crystallinity of any related phase. The size of crystallites of TiO_2_ support has been estimated from the full width at half maximum FWHM of the (110) line of rutile using the classical Scherrer equation [42]. In Table 4, one can see that metal deposition does not produce any significant change in the structure of TiO_2_ support.

Photocatalytic reaction does not induce any noticeable change in the crystal structure of TiO_2_ support powder, as evidenced by the ex situ XRD measurements (Supplementary Material, Figure S4).

#### 2.4.2. SEM Analysis

**Figure 7:**
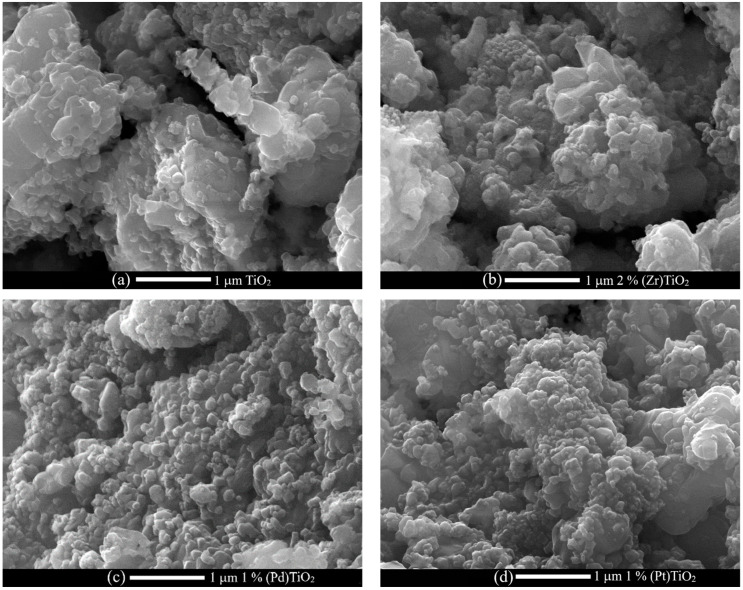
SEM images of representative catalysts (**a**) TiO_2_, (**b**) 2% (Zr)TiO_2_, (**c**) 1% (Pd)TiO_2_, and (**d**) 1% (Pt)TiO_2_ at magnification 25.000×. The percentage 1% or 2% refers to the input molar metal level.

The SEM micrographs of bare TiO_2_ and TiO_2_ decorated with Pt, Pd, and Zr are represented in Figure 7. The micrograph of bare TiO_2_ confirms the existence of larger agglomerates of nanoparticles about 1 micrometer in size, with expressed finer structure features on the surface, in the size range of 20–100 nm-diameter spheres (Figure 7). The EDS spectra are provided in the Supplementary Materials (Figure S1). In summary, morpho-structural characterization reveals no crucial features that could affect catalytic performance, confirming that observed differences in performance originate exclusively from the chemical properties that are tuned by *d*-metal deposition onto the catalyst surface.

### 2.5. Analysis of Degradation Products

**Figure 8:**
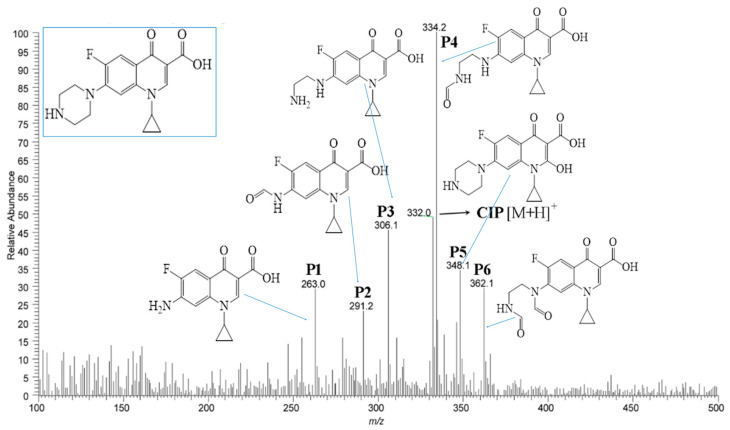
Mass spectrum of the sample after 60 min of CIP photocatalytic degradation. Identified products P1–P6 are represented along with the mass spectrum, while the CIP structural formula is provided as insert (in the blue rectangle).

The degradation products were analyzed for the most efficient catalyst, the 1% (Pt)TiO_2_ photocatalyst. A bare CIP solution subjected to photocatalytic degradation was analyzed by LC-MS/MS. In order to identify degradation byproducts, the sample obtained after 60 min of CIP photocatalytic degradation was selected for mass spectrometry analysis. In the resulting mass spectrum, six degradation products stood out (P1–P6, Figure 8). The precursor ion of each degradation product was then subjected to MS/MS analysis in order to determine the most intensive fragment ion. These fragmentation reactions were subsequently applied for reliable identification of CIP and its degradation products in the samples. A mass chromatogram obtained by LC-MS/MS analysis of the photocatalytic degradation sample after 60 min is shown in Figure S5 of the Supplementary Materials. A mass spectrum with the identified P1–P6 products is shown in Figure 8. A complete list of the identified degradation products P1–P6 with mass spectra, detected fragment ions, and *m/z* ratios is provided in Figure S6 and Table S1 of the Supplementary Materials.

**Figure 9:**
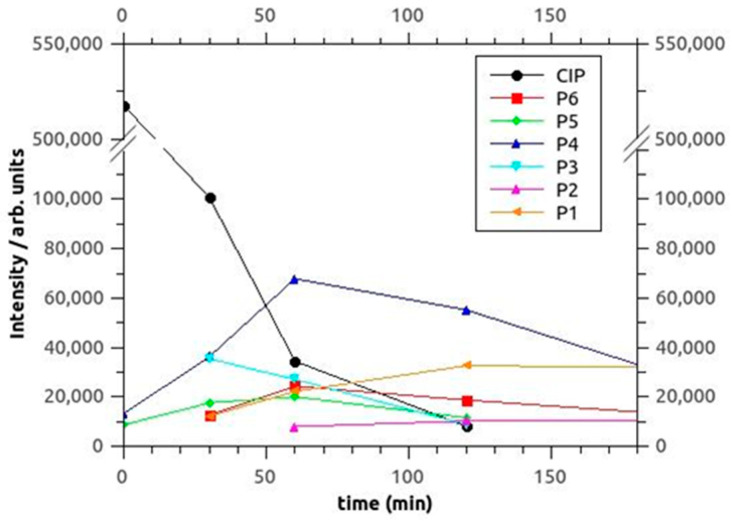
LCMS/MS signal of CIP and degradation products P1–P6 over time.

In addition, the LC-MS/MS signal of P1–P6 was tracked during 240 min of degradation to qualitatively access their formation and depletion trends. The results are represented in Figure 9.

LCMS/MS data confirm the 100% degradation of CIP within the first 120 min of the reaction, while the identified degradation products P1–P6 appear in the degradation mixture within the process course. The increase and decrease in signal is most obvious for P4, confirming that the degradation products are subjected to further depletion.

**Scheme 1:**
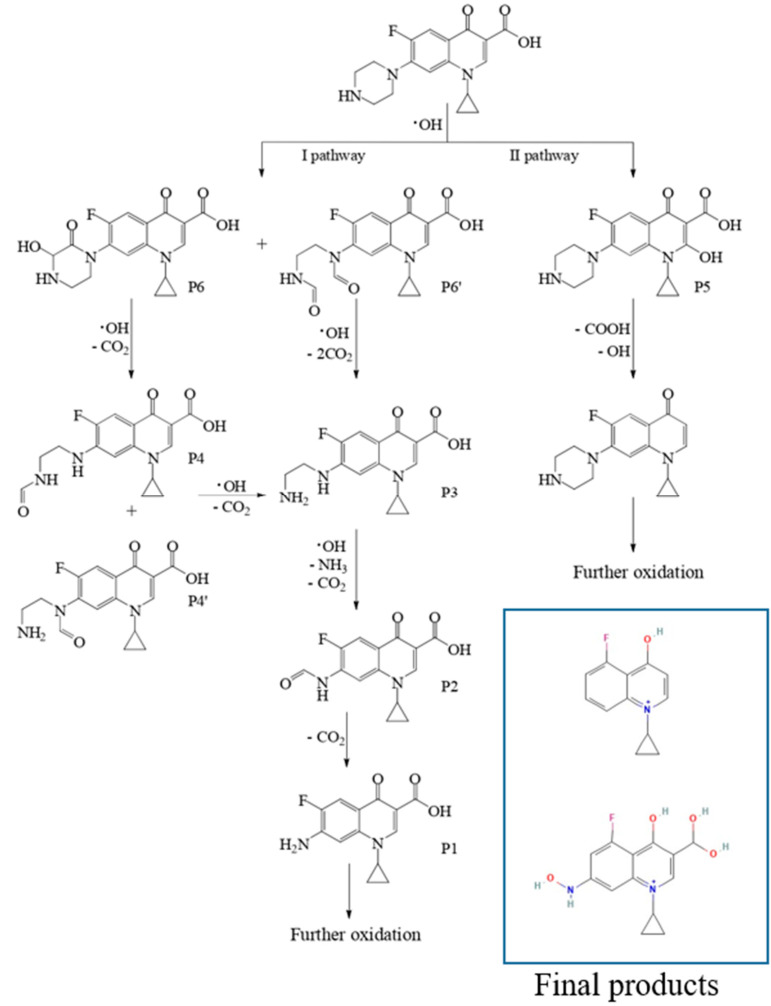
Proposed mechanism of the photodegradation of ciprofloxacin and the proposed residual products after 240 min.

According to previous literature reports, further degradation is expected to undergo defluorination, oxidation, and cycle breaking off, producing highly oxidized aliphatic compounds with many conjugated double bonds, and finally, CO_2_ [43,44,45,46,47]. However, in our study, the mixture of residual ions after 180 min of degradation still includes a significant amount of a variety of ions in the *m*/*z* range of 207–362, with the most intensive signal at *m*/*z* = 279 (see data in Supplementary Material, Table S2), explaining the residual absorbance in the UV-Vis spectra. The similarity of residual UV-Vis spectra to ciprofloxacin and the significant abundance of species with an *m*/*z* ratio over 220 implies that the most resistant structural feature of the degradation products is the fluoro-cyclopropyl-quinoline backbone. Despite the significant depletion of UV-Vis and LC/MS signals, there is no reliable evidence in the present study to indicate the breaking of the fluoro-cyclopropyl-quinoline structure. This is in good agreement with the observation that OH radicals are a main oxidizing species in the system. To achieve the breakage of the structure up to the aliphatic compounds by engaging stronger oxidizing species, one should probably consider using a higher-power lamp and higher-UV energy, introducing additional non-atmospheric oxygen, or using combined methods such as photoelectrodegradation [48]. The proposed reaction mechanism is depicted in Scheme 1.

The detected degradation products P1-P6 identified by LC-MS were analyzed by CompTox chemicals dashboard software (version 3.0.5) [49]. The collected LC_50_ (96-h fathead minnow) and LD_50_ (oral rat), along with developmental toxicity and Ames mutagenicity test values, are represented in Table 5.

The observed results point out that the toxicity of some transition products surpasses CIP. The lowest LD_50_ (691 mg/kg) was observed for P2 – a compound that contains amide groups, and is classified into Class III of the US EPA classification used for pesticides [50]. Also, the Ames mutagenicity test shows decreased mutagenicity compared to CIP for all detected compounds except P5, which shows the same value as CIP. In summary, despite the proven possibility of tuning the kinetics of the process via the deposition of *d*-metals, the degradation byproducts in the photocatalytic process driven by OH radicals persist as a concern due to the limited ability to break the fluoroquinolone backbone. The risk of the formation of small molecules such as formamide and acetamide during the degradation of the piperazine ring also persists as a general concern in AOP applications for the degradation of organic molecules with N-heterocycles. Accordingly, an additional in situ toxicity study is desirable prior to the wider application of the presented methodology. On the other hand, the concentrations of all detected byproducts were much lower compared to the initial ciprofloxacin concentration, and there were no highly toxic products detected by LC-MS/MS analysis.

The mineralization extent was measured with a TOC analysis (Table 6) at the end of the degradation process, for both the ciprofloxacin mixture (used for UV-Vis tracking) and bare ciprofloxacin solution (used for LC-MS/MS). After 240 min of photodegradation with 1% (Pt)TiO_2_, the TOC results show that 24% of the organic carbon was mineralized for the ciprofloxacin solution, and a 16% decrease in organic carbon for the ciprofloxacin mixture was observed. This is in good agreement with previous considerations regarding the stability of the aromatic structures in the CIP molecule in oxidizing conditions.

### 2.6. Comparison of Current Work to Literature Data

Table 7 shows the results of other studies that have investigated ciprofloxacin’s photodegradation with similar photocatalysts. As can be seen from Table 7, the majority of the studies have used high catalyst dosages (200–1500 mg L^−1^), long degradation times, and high-power lamps to achieve degradation extents in the range of 75–100% [14,51,52,53,54]. On the other hand, in the present study, a low-power lamp (0.8 W m^−2^) has been used, and it was designed to assure the reliable UV-Vis tracking of degradation kinetics and to minimize uncontrolled external factors such as chamber heating. Photodegradation with 20 mg of bare TiO_2_ showed an efficiency of around 42% based on the UV-Vis results. Overall, the results are in reasonable agreement with the available literature data on similar catalytic materials, considering the low amount of catalyst compared to the pollutant concentration, the power of the lamps, and the degradation duration.

When centered on metal-modified TiO_2_, the majority of studies presented in Table 7 deal with doped materials and the potential to alternate bulk properties and band gap width.

Some of the works emphasize the multipurpose applicability of the photocatalysts for photocatalytic degradation and hydrogen production, pointing to the need to generalize the knowledge on catalytic processes on the material surface and develop common computational descriptors [37]. Lee et al. [34] have provided an extensive computational and theoretical study of single-atom Cu/TiO_2_, which boosted CO_2_ photoreduction by 66 times. The study emphasizes the catalytic power of single atoms and their ability to tune catalytic activity via the modification of the adsorption energies of reacting species. Our study leans on the above two studies and is the first to deal with the energetics and reactivity of OH radical in photocatalytic degradation, assessing the possibility of tuning it via the deposition of *d*-metals and showing how noticeable the effects it bears are.

Regarding the reaction kinetics, data in the literature offering generalized insights are still lacking—the majority of studies focus on the final efficiency of the process. Our results lean on and corroborate the model of Tran et al. [38], which assumes first-order kinetics for catalytic process where the adsorption/desorption process is the slow step and zero-order kinetics in cases where further depletion processes occurring in the solution are slow. The complexity of the process kinetics was elaborated by Zelkind and Rytwo [55], who reported that the fitted pseudo-order of the photocatalytic degradation of ofloxacin (between 0.26 and 1.20) depends on the input pollutant concentration and the final degradation extent.

**Table 7:** Comparison of current work to literature data.

Catalyst	Catalyst Dosage (mg L^−1^)	Initial CIP Concentration (mg L^−1^)	UV-Irradiation Parameters	Time (min)	Removal Efficiency (%)	Reference
TiO_2_	700	80	24 W λ = 254 nm	600	89	[14]
TiO_2_	1500	33	125 W λ = 365 nm	60	98	[52]
TiO_2_	120	20	100 W λ = 365 nm	60	75	[53]
TiO_2_	250	160	500 W Xenon lamp (CEL-LAX500)	360	96	[56]
TiO_2_	400	500	125 W λ = 254 nm	30	100	[51]
Cu/TiO_2_	20	20	15 W λ = 254 nm	240	50	[24]
Cu-doped AC/TiO_2_	320	100	7 W λ = 254 nm	120	95	[57]
Cu- TiO_2_	500	30	15 W UV-C λ = 254 nm	90	100	[47]
Cu-TiO_2_	250	80	500 W Xenon lamp	240	97	[53]
TiO_2_ P25/Fe^0^	1000	30	10 W λ = 254 nm	60	95	[58]
Fe–N–TiO_2_	20	1000	12 W LED visible light	360	68	[59]
Pt_0_/TiO_2_	1000	5	50 W λ = 450 nm	210	56	[60]
Pt_1.47_/TiO_2_	1000	5	50 W λ = 450 nm	210	86	[60]
PM-TiO_2_-Pd	500	20	10 W LED lamp	240	92	[61]

## 3. Materials and Methods

### 3.1. Preparation of TiO_2_

Titanium (IV)-isopropoxide (Ti(OCH(CH_3_)_2_)_4_) (Sigma-Aldrich, St. Louis, MO, USA, 97%) was dissolved in isopropanol (Centrohem, Stara Pazova, Serbia, 99.5%) and stirred on a magnetic stirrer at room temperature. After 10 min, deionized water (pH = 6) [62] was added dropwise over 3 h. The molar ratio of titanium(IV)-isopropoxide:isopropanol:water was fixed at 5:3:10 [63]. The as-prepared precipitate was washed using ethanol (Reahem, Novi Sad, Serbia, 70%) and dried overnight at 100 °C to remove the moisture and other solvents. Then, it was calcined at 700 °C for 5 h and left in the oven to cool down overnight to attain the rutile phase [64].

### 3.2. Preparation of Zr-, Pt-, Pd-, Fe-, and Cu-Modified TiO_2_

A measure of 500 mg of prepared TiO_2_ nanoparticles was added to 50 mL of deionized water and dispersed using an ultrasonic bath at 90 °C for 30 min. Then, the required amounts (0.5, 1, and 2 at% compared to TiO_2_) of Zr(NO_3_)_4_ (Merck, Darmstadt, Germany, 60% and 40% ZrO_2_), PtCl_4_ (Standard solution, Spectrosol, London, UK), Pd(NO_3_)_2_ (Standard solution, J.T.Baker, Phillipsburg, NJ, USA), FeC_6_H_5_O_7_·5H_2_O (The British Drug House, London, UK, 98%), and Cu(NO_3_)_2_·3H_2_O (Merck, Darmstadt, Germany, 99.5%) and 12.5 mg of solid NaOH (Hemos, Belgrade, Serbia, 99.6%) were added to the mixture and stirred. A measure of 5 mL (0.5, 1, and 2 g L^−1^ for 0.5, 1, and 2% molar ratio, respectively) of NaBH_4_ solution (The British Drug House, London, UK, 95%) was added dropwise to the reaction mixture and stirred at room temperature for 2 h. The suspension was washed, centrifuged, and dried overnight at 100 °C.

### 3.3. Photodegradation of the Ciprofloxacin Infusion Mixture

To start, ciprofloxacin commercial solution for infusion (Marocen^®^, Hemofarm, Serbia), which contains 100 mg of the ciprofloxacin lactate in 10 mL of the solution, was diluted up to the final concentration of 4.75 × 10^−5^ M (20 mg·L^−1^). For LC/MS analysis, ciprofloxacin solution of the same concentration was prepared from pure ciprofloxacin lactate powder (Dr. Ehrenstorfer, Augsburg, Germany, 99%).

The photodegradation procedure was carried out with 20 mg of every catalyst for the ciprofloxacin commercial solution. The volume of the degraded solution was 50 mL. The reaction mixture was stirred for 30 min in the dark, alongside a blank (ciprofloxacin solution without catalyst), and subsequently irradiated under a UV lamp (Philips TUV 15W, with emission maximum at 254 nm, UVC Disinfection, Sroda Wielkopolska, Poland) for 4 h. The measured power of the lamp in experimental conditions was 0.8 W/m^2^. A magnetic stirrer was used throughout the whole degradation period to assure homogenous distribution of the catalyst in the mixture.

Aliquots of 4 mL volume were taken over in intervals of 15–60 min. The photodegradation process was monitored by UV-Vis spectrometry (LLG Labware, Detroit, MI, USA), by recording the UV-Vis spectra in the wavelength range from 190 to 500 nm. To determine ciprofloxacin concentration from UV-Vis absorbance, a calibration plot is recorded and represented at a wavelength of 315 nm (Figure S7, Supplementary Materials, equation: C = 0.1692·A_315_ − 0.013). Degradation efficiency at a given time (t) was calculated from the depletion of ciprofloxacin concentration following the equation:

(4) $$Degradation\;efficiency\left(\%\right)=\frac{C_{\left(t=0\right)}-C_{\left(t\right)}}{C_{\left(t=0\right)}}\times 100\%$$

### 3.4. XRPD Analysis

The X-ray powder diffraction (XRPD) measurements were conducted on Philips PW 1050 X-ray diffractometer using Ni-filtered Cu radiation and Bragg-Brentano focusing geometry. The diffraction intensities were recorded in the 5–70° 2θ range with a step size of 0.02° and a counting time of 6 s per step. Qualitative and semi-quantitative phase analysis has been performed in Difracc. Eva software V7. The unit cell parameters have been extracted from the recorded XRPD patterns by means of Powder Cell software 2.4 [65]. For the estimation of crystallite size, the classical Scherrer method has been applied [42].

### 3.5. SEM-EDS Analysis

Scanning electron microscopy with energy-dispersive spectrometry (SEM-EDS analysis) was performed using a JEOL (Tokyo, Japan) JSM-7001F field emission scanning electron microscope, coupled with an Oxford Instruments (Abingdon, UK) Xplore 15 energy dispersive X-ray spectrometer, in high-vacuum mode (approx. 10^−4^ Pa). The samples were previously coated with a 15 nm thick electrically conductive gold coating. For the SEM imaging, an accelerating voltage of 30 kV, a probe current of approx. 0.5 nA, a working distance of 5 mm, and magnifications of 25,000 and 50,000× were used.

### 3.6. TOC Analysis

Total organic carbon (TOC) was measured on a TOC-LCPH analyzer (Shimadzu Co., Kyoto, Japan). Mineralization efficiency was calculated from Equation (5):

(5) $$Mineralization\;efficiency\left(\%\right)=\frac{{{TOC}_{initial}-TOC}_{final}}{{TOC}_{initial}}\times 100\%$$

### 3.7. LC-MS/MS Analysis

Liquid chromatography was performed using a Dionex UltiMate 3000^®^ LC system (Thermo Fisher Scientific, Waltham, MA, USA). A reverse-phase Zorbax Eclipse^®^ XDB-C18 column, with 75 mm × 4.6 mm i.d. and 3.5 µm particle size (Agilent Technologies, Santa Clara, CA, USA), was used for the separation of the compounds. In front of the chromatographic column, the pre-column was installed, with 12.5 mm × 4.6 mm i.d. and 5 µm particle size (Agilent Technologies). The mobile phase consisted of deionized water (A), methanol (B), and 10% acetic acid (C) (*v*/*v*), and the flow rate was set at 0.6 mL min^−1^. The mobile-phase gradient started with 20% B and 1% C and increased to 40% B within 15 min. The column was then rinsed with 100% B for 5 min, and initial conditions were reestablished within 5 min. Mass spectra were obtained by the LTQ XL (Thermo Fisher Scientific) linear ion trap mass spectrometer. Electrospray was used as the ionization technique in the positive mode. The optimal source parameters were as follows: source voltage, 5 kV; sheath gas, 43 au (i.e., 43 arbitrary units in the 0–100 range defined by the LTQ XL system (Thermo Fisher Scientific, Waltham, MA, USA)); and capillary temperature, 220 °C. Helium was used as a collision gas. Mass spectra were recorded in the *m/z* range of 100–500.

### 3.8. DFT Calculations

For DFT calculations, a pwscf code of the Quantum ESPRESSO package (version 7.2) was used [66]. Ultrasoft pseudopotentials based on GGA-PBE approximation [67] with a plane wave kinetic energy cutoff of 50 eV were implemented, while the charge density cutoff was 900 eV. Optimized rutile bulk parameters were a = 4.639 Å and c = 2.968 Å. In the DFT screening of the adhesion of different *d*-metals, the TiO_2_ surface was modelled as a (110) slab in a 4-layer 1 × 1 (17-atom) cell. There was at least a 25 Å vacuum between slabs, and the dipole correction was employed to avoid interaction between periodic images [68].

All calculations were spin polarized. Hubbard correction (GGA + U) was used in a simplified version of Cococcioni and de Gironcoli’s work [69,70]. An effective U value of 3.0 eV for the Ti-d states, was taken from the literature data [71], and U-corrections were also used for Zr 4d (2.0 eV) and Fe 3d (4.0 eV). The k-point grid was sampled through a Monkhorst–Pack scheme [72], using 4 × 4 × 1 k-points. Electronic and ionic force convergence criteria were 10^−6^ Ry and 10^−4^ Ry/Bohr, respectively. The structures are presented in XcrysDen version 1.6.2-4 [73]. The adhesion of metals was investigated at high-symmetry *bridge* and *top* sites. The adhesion energy on the TiO_2_(110) surface was calculated according to Equation (6):

(6) $$E_{adh}=E_{surf+M}-E_{surf}-E_{M}$$

where E_surf+M_ is the total energy of the surface with adhered metal, E_surf_ is the total energy of the bare TiO_2_(110) surface, and E_M_ is the total energy of the isolated metal atom.

### 3.9. Inductively Coupled Plasma Optical Emission Spectroscopy (ICP-OES)

A measure of 0.1 g of photocatalysts was dissolved in aqua regia, heated, diluted to the concentration 10 g L^−1^, and filtrated. The obtained solutions were analyzed using a Thermo Scientific iCAP 7400 duo ICP-OES. For data acquisition and processing, the Thermo Scientific Qtegra Intelligent Scientific Data Solution (ISDS) software 2.53 was used. For the calibrations, external standards were used, prepared via the dilution of commercial standards (1000 mg L^−1^ Multi-element ICP Standard solution, Chem-Lab, Zedelgem, Belgium; 1 mg·L^−1^ Pd Standard solution, J.T.Baker, Phillipsburg, NJ, USA; 1 mg·L^−1^ Pt Standard solution, J.T.Baker, Phillipsburg, NJ, USA).

## 4. Conclusions

In the present experimental and DFT study, the preparation and optimization of TiO_2_ photocatalyst decorated with a set of *d*-metals commonly used in catalytic processes (Zr, Fe, Cu, Pd and Pt) have been performed in order to drive out calculable theoretical descriptors that can be used in the design of photocatalysts. Based on the proposed reaction models, the experimentally measured degradation rate and efficiency was successfully correlated with the strength of OH radical binding on the metal active site. Accordingly, the high efficiency of (Pt)TiO_2_ and (Pd)TiO_2,_ was attributed to their optimal ability to split water and generate easily reacting OH radicals. The total absorbance depletion was raised from 43 to 61%, and the degradation rate increased by almost double compared to non-modified TiO_2_. The state of the deposited metals on the surface was analyzed experimentally (ICP-OES) and theoretically, revealing that the metal coverage depends on the intrinsic properties of the metal and its input atomic %. Interestingly, the most efficient catalysts, (Pd)TiO_2_ and (Pt)TiO_2_, had low metal coverage of only 1–2 atoms per nm^2^ (single-atom catalysts), further emphasizing the contribution of surface OH radical reactivity and water splitting ability to the photocatalytic performance. The proposed oxidation mechanism via OH radical was confirmed by radical catching experiment in methanol. The understanding of this aspect of photocatalytic degradation opens up novel possibilities for the advanced computational design of catalytic materials.

The analysis of degradation products has revealed a TOC removal of up to 24%. LC-MS/MS analysis confirmed the 100% depletion of ciprofloxacin within the first 120 min. At least six degradation products were detected, and among them, no toxic compounds have been identified. On the other hand, the breakage of the aromatic backbone of the molecule and the formation of aliphatic products has not been evidenced in our experiment, confirming the inability of OH radicals, as the driving oxidant, to attack these highly resistant structures.

## Figures and Tables

**Table 1:** DFT-optimized structures of the TiO_2_(110) surface with adsorbed metal at 0.5 ML coverage, including preferential metal adsorption sites, metal adsorption energies (E_TiO2-M_), and OH adsorption energies on the top metal site (E_ads,OH_).

Metal	Fe	Zr	Cu	Pt	Pd
Top view	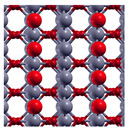	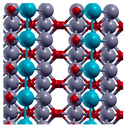	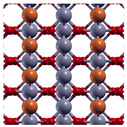	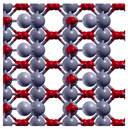	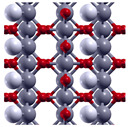
Side view	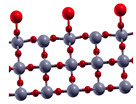	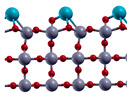	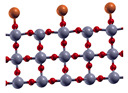	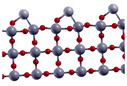	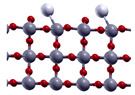
M adsorption site	O-top	Hollow	O-top	Hollow	Hollow
E_M-TiO2_/eV	−3.93	−6.06	−3.99	−4.94	−3.93
E_ads,OH_	−3.67	−5.59	−3.39	−3.21	−2.67

**Table 2:** Experimentally detected values of deposited metal level, calculated coverage, and expected state.

TiO_2_(M)	Fe	Zr	Cu	Pt	Pd
Highest detected metal level (mass %)	0.51 ^1^	1.60 ^2^	1.73 ^1^	0.23 ^1^	0.11 ^1^
Highest estimated coverage (atom nm^−2^)	10–11	20	32	1–2	1–2
Expected state	Fe-O_X_ phase, agglomerated	Zr-O_X_ phase, agglomerated	Cu-O_X_ phase, thin layer	Single atom	Single atom

^1^ ICP-OES measurement, ^2^ SEM-EDS measurement.

**Table 3:** Calculated pseudo-first- and pseudo-zero-order rate constants of CIP degradation and a comparison of degradation rates and degradation efficiency (percentage of CIP degradation after 240 min).

Photocatalyst	k_1_ (min^−1^) × 10^3^	R^2^	k_0_ (mol L^−1^ min^−1^) × 10^3^	R^2^	k(TiO_2_)	CIP Degraded (%)
					k((M)TiO_2_)	
TiO_2_	2.27	0.994	1.74	0.988	1.00	43.74
0.5% (Cu)TiO_2_	3.28	0.981	2.20	0.938	1.44	56.30
1% (Cu)TiO_2_	3.20	0.994	2.23	0.971	1.41	54.16
2% (Cu)TiO_2_	3.06	0.985	2.16	0.976	1.35	54.81
0.5% (Fe)TiO_2_	1.91	0.980	1.09	0.959	0.84	37.26
1% (Fe)TiO_2_	2.01	0.981	1.58	0.974	0.89	39.21
2% (Fe)TiO_2_	2.45	0.991	1.86	0.973	1.08	43.63
0.5% (Zr)TiO_2_	2.01	0.997	1.58	0.993	0.89	39.26
1% (Zr)TiO_2_	1.96	0.996	1.56	0.993	0.86	38.78
2% (Zr)TiO_2_	1.78	0.995	1.44	0.994	0.78	35.09
0.5% (Pd)TiO_2_	3.42	0.998	2.31	0.979	1.51	56.64
1% (Pd)TiO_2_	3.63	0.996	2.41	0.992	1.60	59.67
2% (Pd)TiO_2_	3.21	0.998	2.19	0.982	1.41	52.00
0.5% (Pt)TiO_2_	2.96	0.993	2.08	0.970	1.30	51.72
1% (Pt)TiO_2_	3.87	0.997	2.44	0.930	1.70	61.36
2% (Pt)TiO_2_	3.40	0.997	2.31	0.972	1.50	55.83

**Table 4:** The cell parameters of the TiO_2_ support (rutile; tetragonal s.g. *P*4_2_/*mnm* no. 136) and its crystallite size.

Photocatalyst	Cell Parameters		Xs (nm)
	a (Å)	c (Å)	
TiO_2_	4.595	2.960	71
(Cu)TiO_2_	4.591	2.958	72
(Fe)TiO_2_	4.594	2.959	73
(Pd)TiO_2_	4.594	2.959	68
(Pt)TiO_2_	4.595	2.960	66
(Zr)TiO_2_	4.594	2.960	71

**Table 5:** CompTox values of LC_50_ (96-h fathead minnow), LD_50_ (oral rat), developmental toxicity, and Ames mutagenicity test for detected byproducts of ciprofloxacin photodegradation.

Component Label	Fathead Minnow LC_50_		Oral Rat LD_50_		Developmental Toxicity		Ames Mutagenicity		Toxicity Class	
	−log10 (mol·kg^−1^)	mg·kg^−1^	−log10 (mol·kg^−1^)	mg·kg^−1^	Value	Result	Value	Result	EPA	GHS
CIP	6.24	0.19	1.88	4344	1.09	True	0.62	True	III	V
P1	6.16	0.18	2.38	1099	1.06	True	0.48	False	III	IV
P2	6.36	0.13	2.62	691	1.02	True	0.47	False	III	IV
P3	6.35	0.14	2.05	2699	1.01	True	0.39	False	III	V
P4	6.39	0.13	2.43	1251	1.06	True	0.40	False	III	IV
P4′	6.11	0.26	2.32	1591	1.21	True	0.39	False	III	IV
P5	6.32	0.17	1.95	3887	0.96	True	0.63	True	III	V
P6	6.04	0.33	1.96	3953	0.76	True	0.43	False	III	V
P6′	6.07	0.31	n.a.	n.a.	1.22	True	0.58	True	n.a.	n.a.
fi (*m*/*z* = 288)	6.05	0.26	1.99	2912	0.83	True	0.60	True	III	V

**Table 6:** Results of TOC analysis.

20 mg 1% (Pt)TiO_2_	Initial TOC mg L^−1^ (t = 0 min)	Final TOC mg L^−1^ (t = 240 min)	TOC Removal (%)
CIP mixture	12.41	10.42	16
CIP solution	8.75	6.63	24

## Data Availability

All data are provided in the manuscript or Supplementary information. Additional data will be provided by the authors on request.

## Supplementary Materials

The supporting information can be downloaded at: https://www.mdpi.com/article/10.3390/ijms252111844/s1.
