# Supplementary material for: Uncovering Metal-Decorated TiO2 Photocatalysts for Ciprofloxacin Degradation—A Combined Experimental and DFT Study
Source: Int J Mol Sci. 2024 Nov 4;25(21):11844. doi: 10.3390/ijms252111844 (PMC11546676; doi:10.3390/ijms252111844)
Supplement: Supplementary file 1 [file ijms-25-11844-s001.zip › ijms-3257989-supplementary.pdf]

S1. Coverage estimation method

The metal coverage of TiO_2 (ϴ) was estimated as the number of metal atoms per square angstrom of TiO_2 surface. The molar TiO_2 surface was assumed as the total surface of 1 mol of TiO_2 if consisted of spheres of 50 nm in diameter.

$$\Theta \approx m_{metal} /(S_{TiO2} *M_{metal})= (4/3*Pi*R^3*d_{TiO2}*C_{ICP\text{-}OES})*N_A/(4*R^2*Pi*M_{metal})$$

$$\Theta \approx 4/3*R* d_{TiO2}*C_{ICP\text{-}OES}/M_{metal} \qquad \text{(Eq. S-1)}$$

where R is the average radius of a TiO_2 sphere estimated from SEM micrographs (assumed 25 nm), d_{TiO2} is the tabular value of TiO_2 rutile density (4.23 g/cm^3) , $C_{ICP\text{-}OES}$ – ICP-OES measured mass concentration of metal in TiO_2 sample (g/L), M_{metal} – metal molar mass, N_A – Avogadro number

S2. EDS spectra of the investigated samples

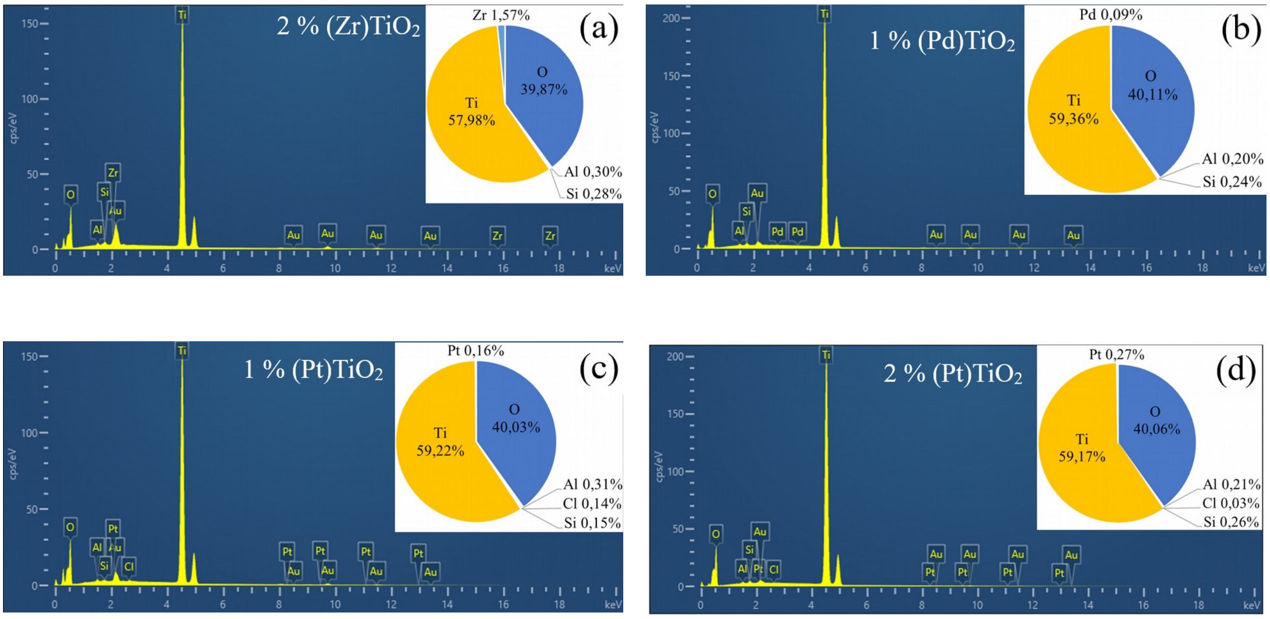

Figure S1. SEM-EDS spectra and normalized amount of the elements from EDS analysis: (a) 2 % $(Zr)TiO_2$, (b)1 % $(Pd)TiO_2$, (c) 1 % $(Pt)TiO_2$ and (d) 2 % $(Pt)TiO_2$.

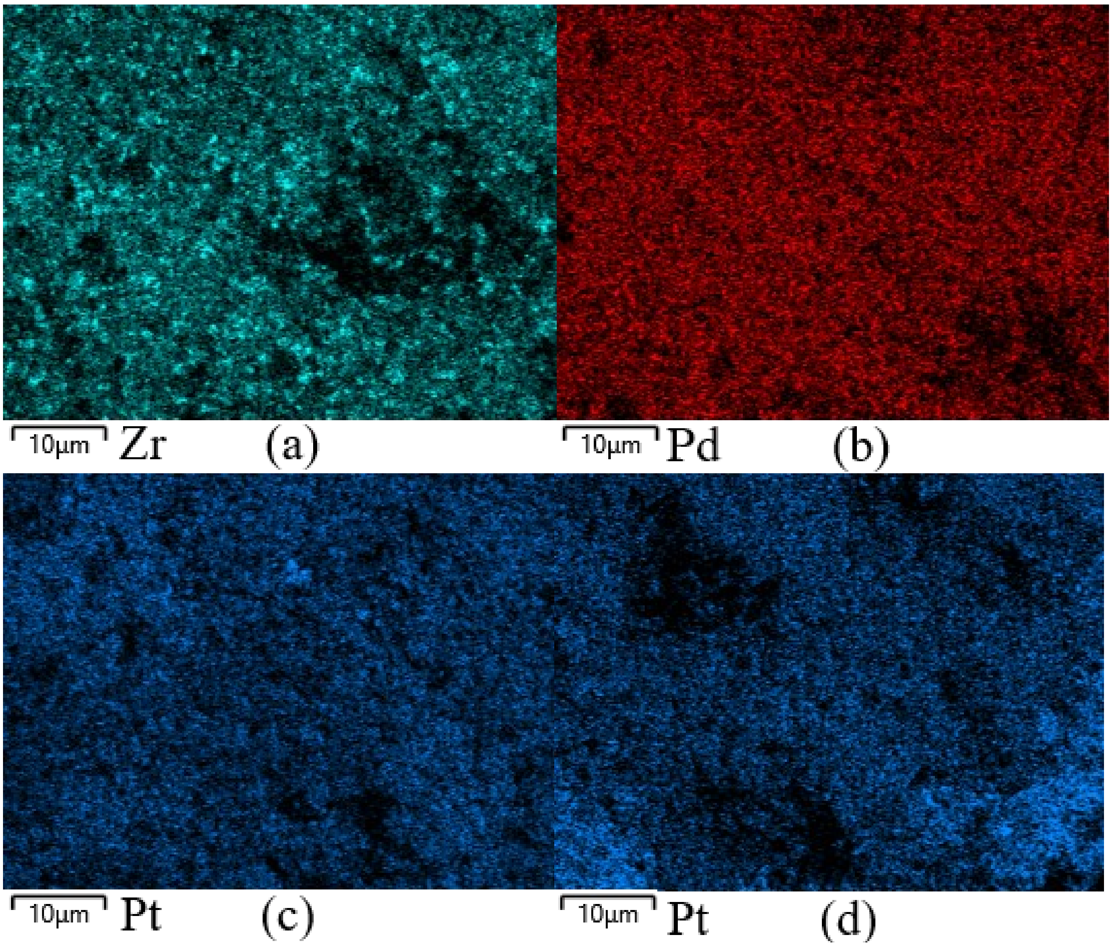

Figure S2. Elemental maps and distribution of metals (a) 2 % (Zr)TiO_2, (b) 1 % (Pd)TiO_2, (c) 1 % (Pt)TiO_2 and (d) 2 % (Pt)TiO_2.

S3. UV-Vis spectra of ciprofloxacin degradation with 20 mg of photocatalyst – all materials

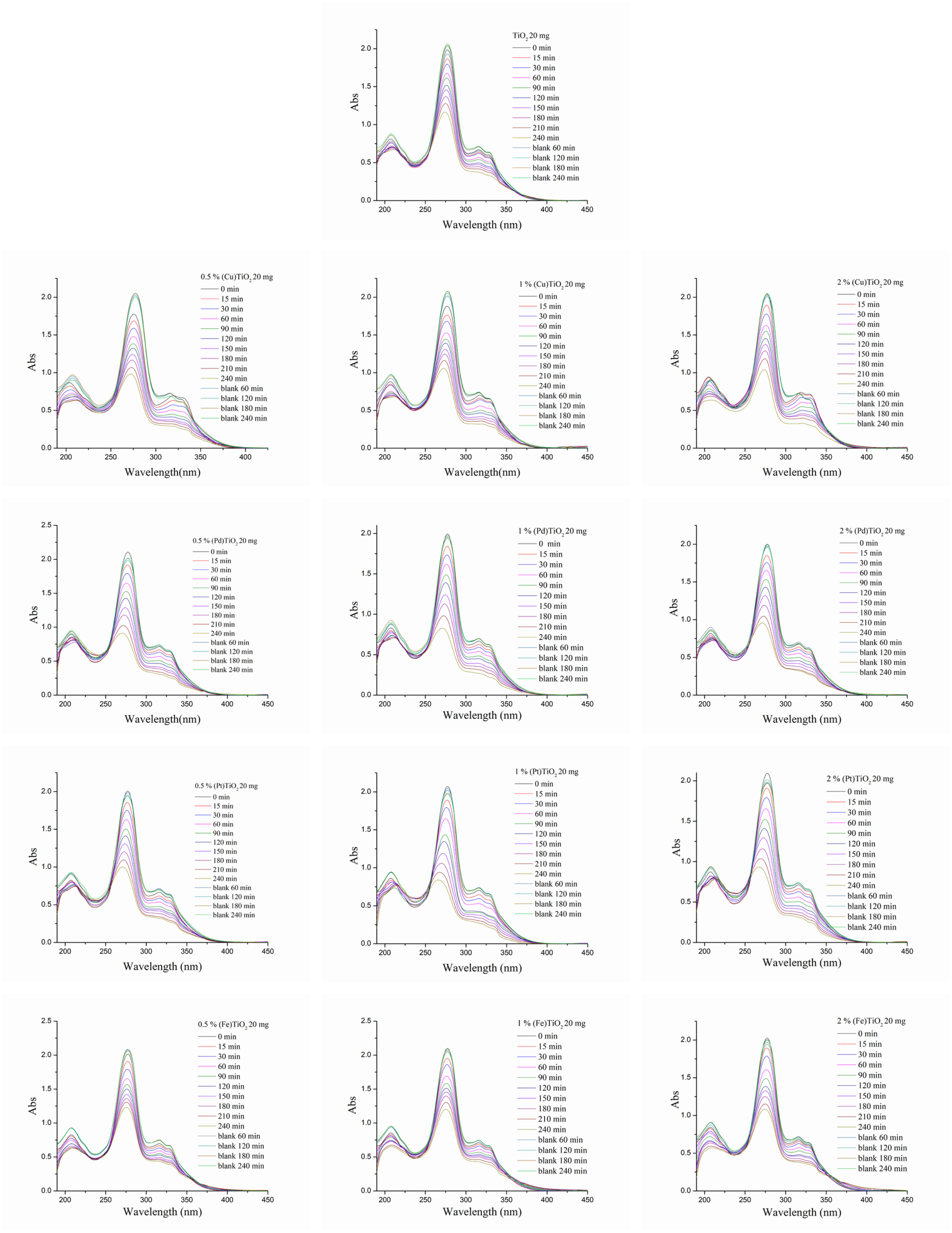

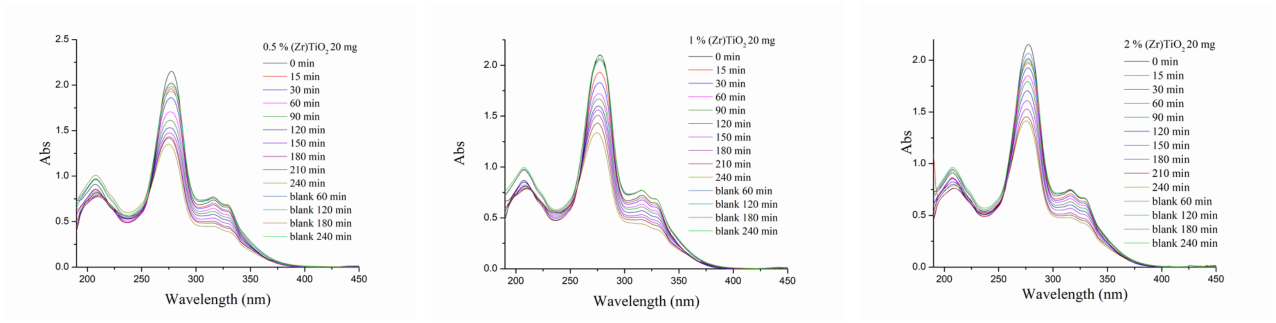

a)

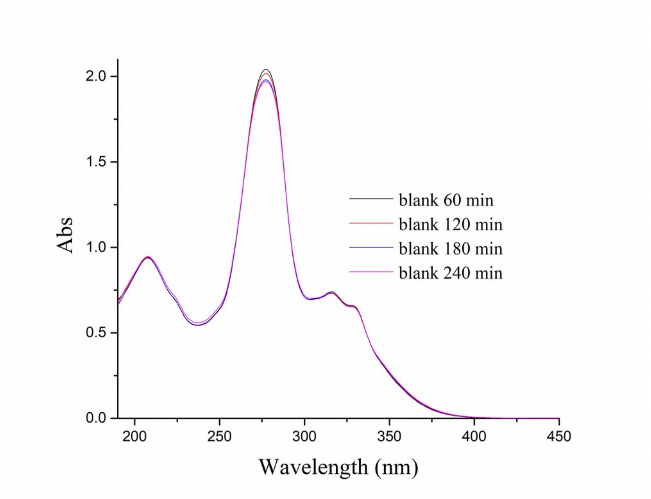

b)

Figure S3. a) UV-Vis spectra of degradation process with 20 mg of catalyst for bare TiO_2 and investigated modified catalysts (Pd, Pt, Zr, Cu, Fe - TiO_2) b)UV-Vis spectra of the irradiated blank solution (CIP without photocatalyst) within 240 min.

S4. XRD stability check

Photocatalytic reaction does not induce any noticeable change in the crystal structure of TiO_2 support powder as evidenced by the *ex situ* XRD measurements (Figure S1).

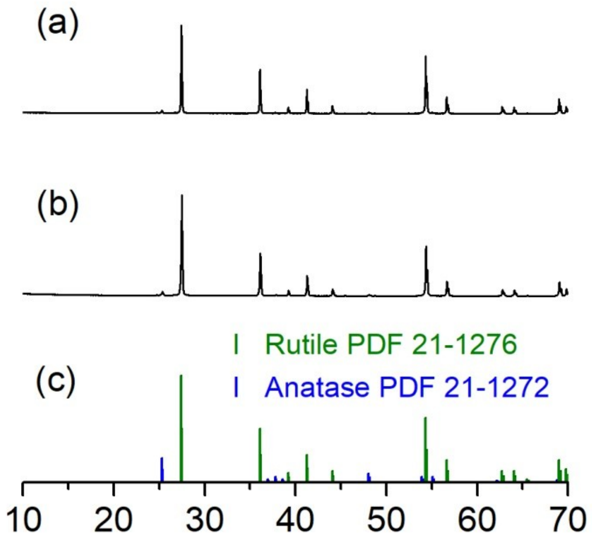

Figure S4. XRD pattern of the (Pt)TiO_2 after photocatalytic reaction (a), as prepared (Pt)TiO_2 (b) and reflections of the corresponding PDF cards (c).

S5. A mass chromatogram P1 - P6

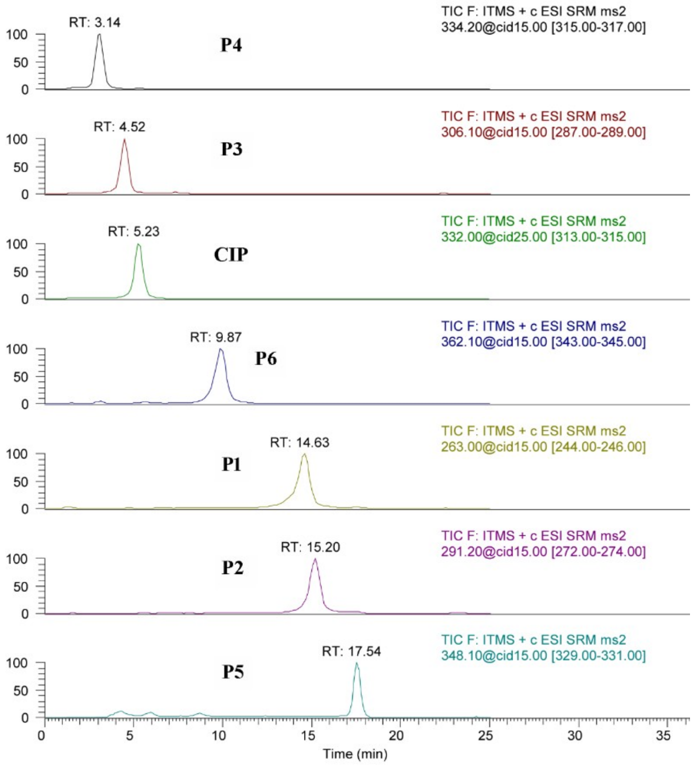

Figure S5. A mass chromatogram of the photocatalytic degradation samples after 60 min of photodegradation.

S-6. Mass spectra of degradation prodcuts P1-P6

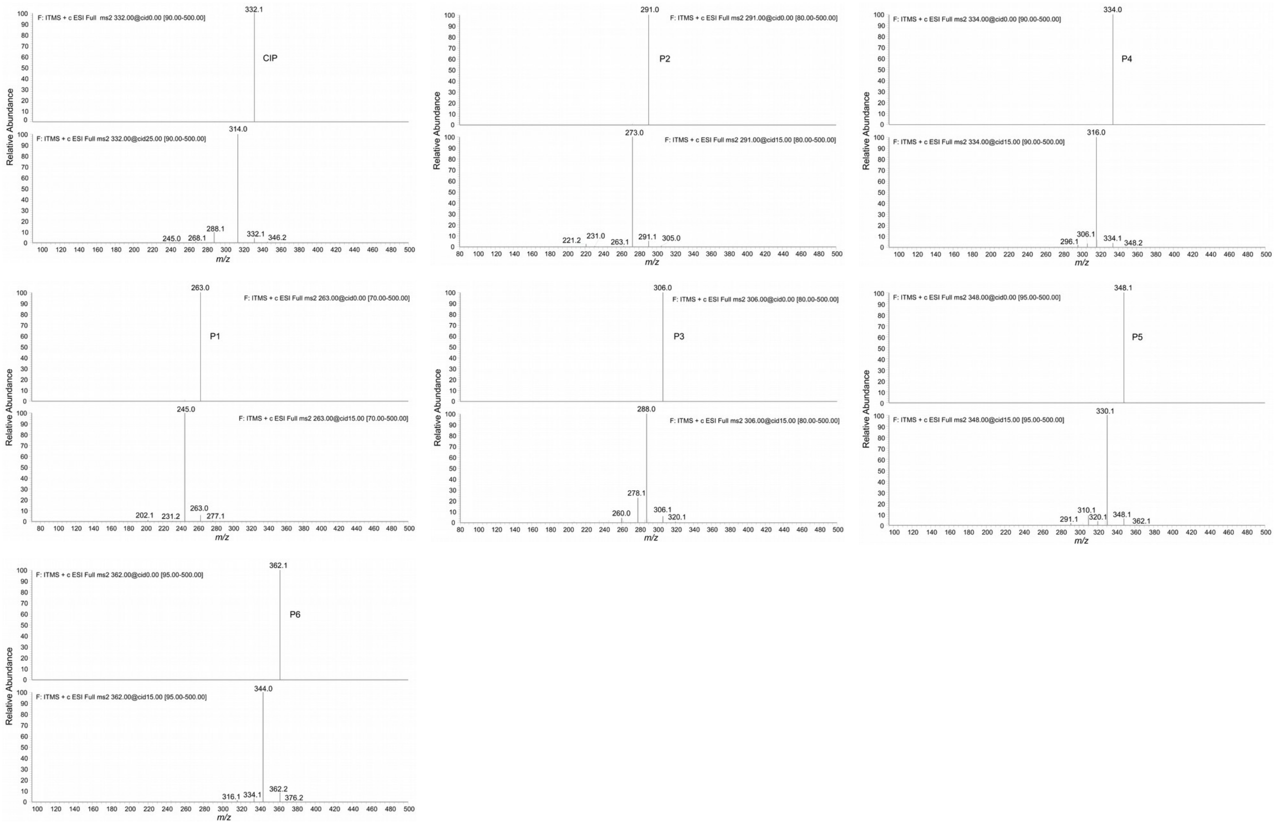

Figure S6. Mass spectra photocatalytic degradation products P1-P6 after 60 min of photodegradation.

Table S1. CIP photocatalytic degradation products and their MS/MS fragment ions

Component formula	Precursor ion (*m/z*)	Fragment ions (*m/z*)	Proposed structure
CIP<br>$C_{17}H_{18}FN_3O_3$	332 $[M+H]^+$	314, 288, 268, 245, 346	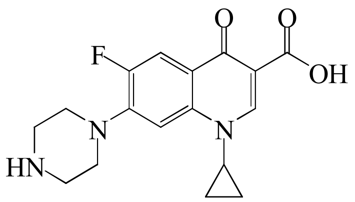
P1<br>$C_{13}H_{11}FN_2O_3$	263	245, 277, 202, 231	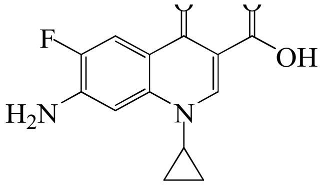
P2<br>$C_{14}H_{11}FN_2O_4$	291	273, 263, 231, 221, 305	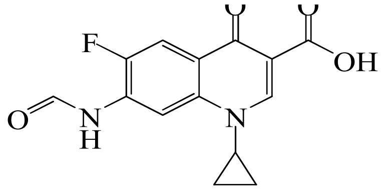
P3<br>$C_{15}H_{16}FN_3O_3$	306	288, 278, 320, 260	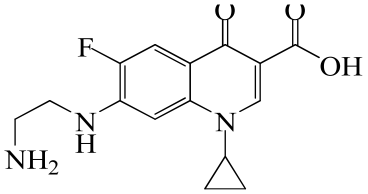
P4<br>$C_{16}H_{16}FN_3O_4$	334	348, 316, 306, 296	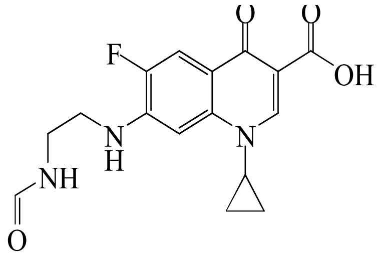<br>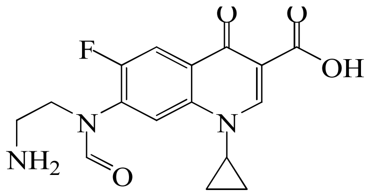

P5<br>$C_{17}H_{18}FN_3O_4$	348	362,330, 310,<br>320, 291	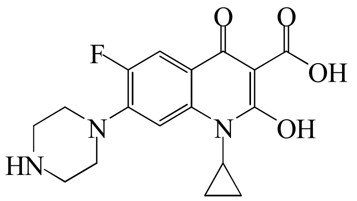<br>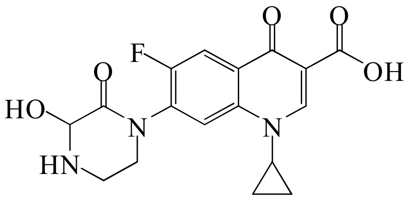
P6<br>$C_{17}H_{16}FN_3O_5$	362	344, 334, 316,<br>376	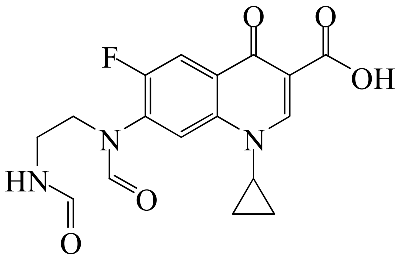

S7. LCMS/MS raw data

Table S2. Measured LCMS/MS signal intensities for the degradation times 0- 240 min.

C:\Xcalibur\...\CPI_uz_0min_u_MF_50B1
protok 15 ul/min

CPI_uz_0min_u_MF_50B1C#2-184
F: ITMS + c ESI Full ms [90.(
m/z= 200.00-500.00

m/z	Intensity	Relative
206.96	13528.3	2.61
210.98	6884.3	1.33
214.92	14941.7	2.89
216.98	11763.7	2.27
218.93	24360.6	4.71
220.97	13831.9	2.67
222.97	24828.0	4.80
235.04	8824.5	1.70
238.05	6355.4	1.23
238.99	17040.3	3.29
246.93	48894.0	9.45
248.94	29566.0	5.71
250.07	9235.9	1.78
250.97	72092.6	13.93
252.03	7660.9	1.48
252.98	8493.0	1.64
254.95	19281.6	3.72
266.98	9291.9	1.80
274.83	16156.4	3.12
277.00	8913.0	1.72
279.03	65758.8	12.70
280.11	11158.6	2.16
281.04	7274.3	1.41
282.88	48593.7	9.39
287.01	8521.5	1.65
296.14	14180.0	2.74
301.10	10326.8	2.00
310.90	25756.8	4.98
314.83	13513.4	2.61
332.09	517628.8	100.00
333.11	99816.5	19.28
334.15	13178.1	2.55
348.16	8667.2	1.67
354.12	10954.4	2.12
376.34	6887.2	1.33

t=0 min

CIP_uz30min_u_MF_30B1C_mz_200-5
protok 15 ul/min

CIP_uz30min_u_MF_30B1C_mz_2(
F: ITMS + c ESI Full ms [200

m/z	Intensity	Relative
206.98	12651.5	12.56
211.00	8733.7	8.67
214.99	9702.8	9.63
217.02	13973.3	13.87
218.97	14837.5	14.73
220.98	28730.7	28.51
222.98	27393.6	27.19
224.25	10901.8	10.82
225.03	43306.5	42.98
227.07	8490.0	8.43
238.99	15568.8	15.45
246.86	24206.7	24.02
249.00	15940.5	15.82
250.97	46645.3	46.29
253.03	11671.0	11.58
255.01	13473.1	13.37
257.07	15313.0	15.20
263.04	12155.9	12.06
274.27	10444.5	10.37
275.08	15210.2	15.10
279.05	55554.0	55.13
280.10	9791.0	9.72
282.97	21107.3	20.95
296.15	9742.0	9.67
301.10	10166.2	10.09
306.06	35422.7	35.16
307.10	11463.0	11.38
310.94	10380.5	10.30
330.11	8996.8	8.93
332.07	100760.5	100.00
333.13	22682.0	22.51
334.10	36241.9	35.97
335.10	8359.4	8.30
348.09	17532.1	17.40
362.09	12533.2	12.44

t=30 min

CPI_uz_60min_u_MF_50B1C_DP 291
protok 15 ul/min

CPI_uz_60min_u_MF_50B1C_DP 2!
F: ITMS + c ESI Full ms [200.

m/z	Intensity	Relative
206.98	13234.7	16.08
214.93	15692.5	19.07
216.93	10304.1	12.52
218.91	27762.0	33.73
220.95	12406.5	15.07
222.97	25699.6	31.23
235.04	9321.0	11.33
238.98	11970.5	14.54
246.90	56717.4	68.92
248.93	33254.6	40.41
250.07	9463.3	11.50
250.94	82300.0	100.00
252.01	8609.7	10.46
252.97	7105.3	8.63
254.80	21972.0	26.70
263.02	22158.2	26.92
266.97	7492.6	9.10
274.82	18557.7	22.55
276.98	10519.0	12.78
278.99	49033.3	59.58
280.08	7870.8	9.56
282.85	49965.3	60.71
291.06	7900.5	9.60
306.10	27012.4	32.82
307.09	7128.0	8.66
310.85	26420.6	32.10
330.13	7315.3	8.89
332.11	34409.4	41.81
333.21	9469.0	11.51
334.09	67798.4	82.38
335.10	13618.3	16.55
346.13	10699.1	13.00
348.07	20036.1	24.35
362.10	24302.3	29.53
366.15	8011.4	9.73

t=60 min

C:\Xcalibur\...\CPI_uz_120min_u_MF_50E
protok 15 ul/min

CPI_uz_120min_u_MF_50B1C#6-25
F: ITMS + c ESI Full ms [90.0
m/z= 200.00-500.00

m/z	Intensity	Relative
206.98	26897.2	28.05
214.91	13466.4	14.04
217.00	11747.0	12.25
218.94	21929.4	22.87
220.96	13669.8	14.26
222.96	35306.8	36.82
235.00	17430.4	18.18
238.97	18730.3	19.53
246.92	44721.2	46.64
248.94	28353.0	29.57
250.09	8731.6	9.11
250.94	95889.2	100.00
252.03	10849.6	11.31
252.99	9414.4	9.82
254.93	28999.2	30.24
263.04	32574.8	33.97
267.00	10339.3	10.78
275.00	9069.7	9.46
277.01	9130.1	9.52
279.03	81449.0	84.94
280.10	14306.7	14.92
281.02	8312.0	8.67
282.87	73506.8	76.66
283.99	8739.0	9.11
285.01	7888.5	8.23
287.00	11093.9	11.57
291.03	10220.7	10.66
296.13	18297.1	19.08
301.09	11383.5	11.87
306.11	8868.4	9.25
310.84	38008.5	39.64
314.40	9524.8	9.93
315.00	13366.8	13.94
324.19	10224.4	10.66
332.15	8135.2	8.48
334.09	55198.2	57.56
335.13	11800.0	12.31
339.05	8145.2	8.49
348.16	11523.8	12.02
362.12	18689.3	19.49

t=120 min

C:\Xcalibur\...\CPI_uz_180min_u_MF_50
protok 15 ul/min

CPI_uz_180min_u_MF_50B1C#8-4
F: ITMS + c ESI Full ms [100.
m/z= 200.00-500.00

m/z	Intensity	Relative
206.97	15272.0	18.92
210.97	8530.1	10.57
214.93	13503.6	16.73
216.99	11697.4	14.49
218.38	9200.8	11.40
218.95	21743.1	26.93
220.95	16601.9	20.57
222.95	30474.9	37.75
224.99	8187.3	10.14
235.04	9284.0	11.50
238.99	15658.7	19.40
246.91	42466.8	52.61
248.96	27043.7	33.50
250.07	9067.2	11.23
250.94	75767.9	93.86
252.03	9942.3	12.32
252.97	9944.2	12.32
254.39	10724.3	13.29
254.98	21149.5	26.20
263.03	31764.1	39.35
274.83	13888.1	17.20
277.01	8432.4	10.45
279.04	80724.5	100.00
280.10	15275.8	18.92
281.03	8490.3	10.52
282.84	55533.2	68.79
286.99	11622.4	14.40
291.06	10130.9	12.55
296.12	23534.7	29.15
301.09	12821.4	15.88
310.80	26180.4	32.43
314.99	11190.8	13.86
324.21	12830.5	15.89
334.12	33082.6	40.98
362.11	13877.6	17.19

t=180 min

C:\Xcalibur\...\CIP_uz240min_u_struji_M
protok 15 ul/min

CIP_uz240min_u_struji_MF#3-
F: ITMS + c ESI Full ms [20(

m/z	Intensity	Relative
202.94	271.4	3.90
207.03	769.0	11.04
209.04	306.6	4.40
210.00	384.1	5.52
211.00	499.4	7.17
212.95	1395.4	20.04
218.95	849.8	12.20
220.97	501.5	7.20
222.98	6695.1	96.16
224.01	507.8	7.29
225.02	606.8	8.72
226.99	6962.4	100.00
228.04	599.6	8.61
229.03	989.5	14.21
230.15	305.1	4.38
231.04	327.3	4.70
239.08	354.7	5.10
244.97	1655.6	23.78
249.06	242.6	3.48
251.04	351.7	5.05
255.00	1088.7	15.64
262.96	641.6	9.22
269.00	325.3	4.67
279.10	1243.8	17.86
294.99	686.2	9.86
301.11	996.9	14.32
305.08	327.9	4.71
321.01	892.7	12.82
344.99	836.7	12.02
353.07	315.7	4.53
370.99	751.1	10.79
409.16	279.5	4.01
413.21	341.1	4.90
420.97	534.0	7.67
427.02	432.6	6.21

t=240 min

S8. UV -Vis calibration plots

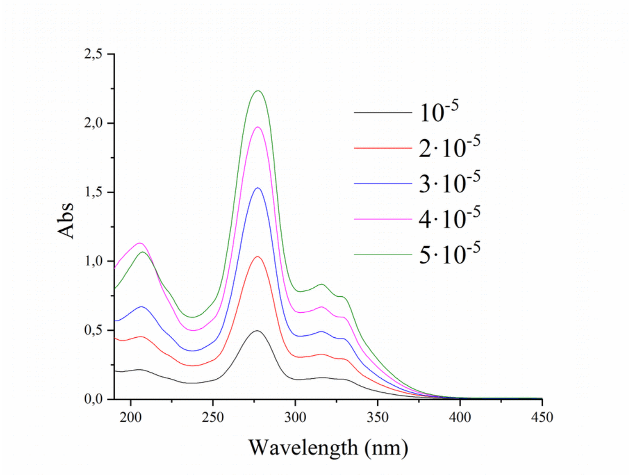

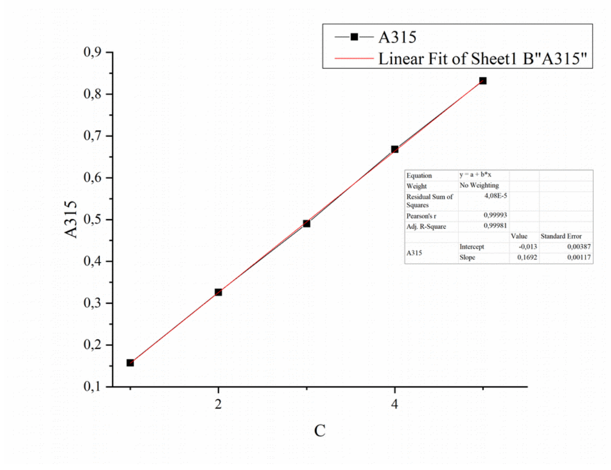

Figure S7: left -UV Vis spectra of commercial CIP solution in the concentration range 1 – 5x10^{-5} M and right – calibration plot at 315 nm
